# Intestinal mRNA expression profiles associated with mucosal healing in ustekinumab-treated Crohn's disease patients: bioinformatics analysis and prospective cohort validation

**DOI:** 10.1186/s12967-024-05427-w

**Published:** 2024-06-26

**Authors:** Qing Li, Zicheng Huang, Hongsheng Yang, Jian Tang, Tao Zuo, Qingfan Yang, Zhaopeng Huang, Qin Guo, Miao Li, Xiang Gao, Kang Chao

**Affiliations:** 1 Department of Gastroenterology, The Sixth Affiliated Hospital, Sun Yat-Sen University, No.26 Yuancun Road II, Tianhe District, Guangzhou, 510000 People’s Republic of China; 2 Guangdong Provincial Key Laboratory of Colorectal and Pelvic Floor Diseases, the Sixth Affiliated Hospital, Sun Yat-Sen University, Guangzhou, People’s Republic of China; 3 Biomedical Innovation Center, The Sixth Affiliated Hospital, Sun Yat-Sen University, Guangzhou, People’s Republic of China; 4 Key Laboratory of Human Microbiome and Chronic Diseases, Ministry of Education, Sun Yat-Sen University, Guangzhou, People’s Republic of China; 5 Biomedical Innovation Centre, The Sixth Affiliated Hospital, Sun Yat-Sen University, Guangzhou, People’s Republic of China

**Keywords:** Crohn's disease, Ustekinumab, LASSO regression, KDM5D, LCN2

## Abstract

**Background:**

Variations exist in the response of patients with Crohn’s disease (CD) to ustekinumab (UST) treatment, but the underlying cause remains unknown. Our objective was to investigate the involvement of immune cells and identify potential biomarkers that could predict the response to interleukin (IL) 12/23 inhibitors in patients with CD.

**Methods:**

The GSE207022 dataset, which consisted of 54 non-responders and 9 responders to UST in a CD cohort, was analyzed. Differentially expressed genes (DEGs) were identified and subjected to Gene Ontology (GO) and Kyoto Encyclopedia of Genes and Genomes (KEGG) pathway analyses. Least absolute shrinkage and selection operator (LASSO) regression was used to screen the most powerful hub genes. Receiver operating characteristic (ROC) curve analysis was performed to evaluate the predictive performances of these genes. Single-sample Gene Set Enrichment Analysis (ssGSEA) was used to estimate the proportions of immune cell types. These significantly altered genes were subjected to cluster analysis into immune cell-related infiltration. To validate the reliability of the candidates, patients prescribed UST as a first-line biologic in a prospective cohort were included as an independent validation dataset.

**Results:**

A total of 99 DEGs were identified in the integrated dataset. GO and KEGG analyses revealed significant enrichment of immune response pathways in patients with CD. Thirteen genes (SOCS3, CD55, KDM5D, IGFBP5, LCN2, SLC15A1, XPNPEP2, HLA-DQA2, HMGCS2, DDX3Y, ITGB2, CDKN2B and HLA-DQA1), which were primarily associated with the response versus nonresponse patients, were identified and included in the LASSO analysis. These genes accurately predicted treatment response, with an area under the curve (AUC) of 0.938. T helper cell type 1 (Th1) cell polarization was comparatively strong in nonresponse individuals. Positive connections were observed between Th1 cells and the LCN2 and KDM5D genes. Furthermore, we employed an independent validation dataset and early experimental verification to validate the LCN2 and KDM5D genes as effective predictive markers.

**Conclusions:**

Th1 cell polarization is an important cause of nonresponse to UST therapy in patients with CD. LCN2 and KDM5D can be used as predictive markers to effectively identify nonresponse patients.

*Trial registration*: Trial registration number: NCT05542459; Date of registration: 2022-09-14; URL: https://www.clinicaltrials.gov.

**Supplementary Information:**

The online version contains supplementary material available at 10.1186/s12967-024-05427-w.

## Background

Crohn's disease (CD) is a chronic inflammatory disease of the gastrointestinal tract that requires long-term therapy to control symptoms and prevent progression [1, 2]. The exact cause of CD is unknown, with complex cross-talk of immune system, genetics, environmental factors, and the microbiome [3]. Recent developments have improved our understanding of the pathogenesis of CD, notably by unraveling the underlying genetic risk factors [4], the aberrant immune response [5], and dysregulation of the gut microbiome and metabolome [6, 7]. Intestinal T helper cell type 1 (Th1) cells are key immune cells in the maintenance of intestinal immune homeostasis [8]. Disruption of mucosal immunity plays a critical role in the pathogenesis of CD, yet its mechanism remains not fully elucidated.

Over the past two decades, treatment targets for CD have evolved from clinical to endoscopic remission [9]. Responses to the new therapies, including biologics and small molecular drugs, are highly heterogeneous, which brings the concept of personalized therapy” into the spotlight [10]. Efforts have been made to guide personalized therapies from a genetic perspective. Patients with CD with a NOD2 gene mutation exhibit a distinct clinical phenotype and require higher doses of anti-tumor necrosis factor (TNF)-α agents to attain adequate anti-TNF-α trough levels [11]. Studies have reported an independent association between HLADQA1*05 and an increased risk of developing antibodies against infliximab in patients with IBD [12, 13]. West et al. [14] identified a newly implicated cytokine, oncostatin M (OSM), its receptor (OSMR), and a co-expressed transcriptional module as predictors of nonresponse to anti-TNF therapy. Tissue transcriptomics have also been used to predict responses to anti-integrin therapy [15]. These data highlight the importance of understanding the mucosal immunopathology in IBD and its relationship with drug response prediction.

Ustekinumab (UST) has recently gained global use and is considered an ideal therapy because of its high safety profile for treating inflammatory conditions such as psoriasis, psoriatic arthritis, and CD [16, 17]. UST is a monoclonal antibody that targets the p40 subunit of interleukin (IL)-12 and -23. IL-12 is important for Th1 differentiation, while IL-23 is involved in the T helper cell type 17 (Th17) pathway [18]. Theoretically, UST can modulate both Th1 and Th17 cell responses, thereby reducing inflammation and inhibiting the development of inflammatory T cells [19]. Both clinical trials and real-world studies have confirmed the effectiveness and safety of UST, although therapeutic responses to UST have only been observed in a specific subset of patients [18, 20–24]. However, variations in patient responses to UST treatment may be influenced by several factors, including the involvement and characteristics of different immune cells [24]. Predicting responses to UST is not well studied. Only one study has explored predictors of primary nonresponders in patients with CD receiving UST therapy [25]. However, this study did not directly identify differential genes between UST responders and nonresponders. In addition, the immune mechanisms underlying the variations involved in nonresponse to UST in patients with CD also remained elusive.

This study aimed to identify appropriate biomarkers for guiding clinical practice. We comprehensively explored the gene differences between responders and primary nonresponders to UST induction therapy for CD using the Gene Expression Omnibus (GEO) database. This prospective cohort was conducted to validate the findings from our discovery cohort. We then verified hub gene expression in UST clinical responders compared to that in nonresponders.

## Methods

### Study design and patient population

Our study design incorporated independent discovery and validation datasets. We used the GSE207022 dataset obtained from the GEO public database (http://www.ncbi.nlm.nih.gov/geo/), with the annotation platform GPL13158 as the discovery dataset. For validation, we included patients with CD who received UST therapy and were enrolled in the prospective registry of the MORE study (NCT05542459) between July 1, 2022, and July 1, 2023, at the Sixth Affiliated Hospital, Sun Yat-sen University. The inclusion criteria were as follows: individuals diagnosed with CD who received UST treatment and patients who were administered an initial intravenous dose of UST based on their weight for induction therapy, followed by at least one 90 mg subcutaneous maintenance injection of UST as the initial treatment. Patients were excluded if they had other factors that could complicate the results, such as concurrent infection, short bowel syndrome, or any condition requiring surgery; were taking other biological medications; or had incomplete data. Intestinal inflammation activity was evaluated based on the Simplified Endoscopic Score of Crohn’s Disease (SES-CD). Endoscopic biopsy samples were collected from terminal ileum at baseline for mRNA sequencing. All patients underwent colonoscopy at week 24 from baseline to follow-up.

### Transcriptome sequencing

Twenty-two endoscopic biopsy samples were collected for RNA-Seq. (1) Library construction: Total RNA was obtained from Guangdong Meg Gene Biotechnology Co., LTD. (Guangzhou, China) using a commercial kit and according to the manufacturer's instructions. RNA degradation and contamination were detected by 1% agarose gel electrophoresis. Both Qubit4.0 (Thermo Fisher Scientific, Waltham,MA, USA) and Nanodrop One (Thermo Fisher Scientific) were used to quantify the RNA. The RNA integrity was determined using an Agilent 4200 system (Agilent Technologies, Santa Clara, CA, USA). (2) Library construction: The library was built using the ALFA-SEQ RNA Library Prep Kit as recommended by the manufacturer according to the manufacturer’s instructions. (3) Sequencing: Library sequencing on the Illumina or MGI platforms produced a paired-end reading of 150 bp.

### Data acquisition and pre-processing

The GSE207022 dataset contained samples from 125 patients with moderately to severely active CD and 23 healthy controls. Patients treated with intravenous UST who underwent endoscopic assessment at week 8 were included in the analysis. UST‐ induced responders were defined as those with mucosal healing with SES-CD scores < 3 [26]. We included sixty-three patients with complete profiles and clinical information, including the response (n = 9) and nonresponse (n = 54) arms. The “handout” method was used for splitting samples in the nonresponse arm. Fifty-four samples were randomly divided into six groups (n = 9 per group) using the caret package (version 6.0–85).

### Analysis of differentially expressed genes (DEGs)

Principal component analysis (PCA) was used to compare and visualize the within-group sample consistency. DEGs were analyzed using the GEO2R for the responder and nonresponder groups in the GSE207022 dataset (http://www.ncbi.nlm.nih.gov/geo/geo2r). The cut‐off values for significant DEGs were |log2(FC)|> 1 and p-value < 0.05. DEGs were visualized with volcano plots using the ggplot R package. An upset diagram was used to represent the intersection between the DEGs and groups.

### GO and KEGG function analysis

ClusterProfiler was used to annotate the functional aspects of diverse genes to explore their functional significance. GO and KEGG were used to evaluate related functional classifications. Pathways enriched in GO and KEGG analyses with adjusted p < 0.05 were considered significant.

### Construction of least absolute shrinkage and selection operator (LASSO) regression model

LASSO regression was used to identify key genes associated with UST response. The gene expression data were integrated into a regression coefficient-weighted scoring formula, to develop a predictive model for patients. We evaluated the predictive accuracy of the model using receiver operating characteristic (ROC) curves.

### Univariate logistic analysis

Significant DEGs were analyzed using univariate logistic regression to investigate their association with the UST response. This analysis was conducted using R studio by fitting a generalized linear model with the main argument "family = binomial." Subsequently, hazard ratio (HR), 95% confidence interval (95% CI), and p-values were computed. The univariate logistic regression analysis results were presented as a forest plot using the "forestplot" R package (version 1.9).

### Immune cell infiltration analysis by ssGSEA

Using the ssGSEA algorithm, a reliable tool for determining cell composition, we calculated the proportions of immune cell types present in the intestinal tissues based on gene expression profiles. The ssGSEA method was implemented using the ssGSEA function to calculate the scores of immune infiltration for 24 immune cell types in the R package “GSVA” [27, 28]. Pearson’s correlation analysis was used to evaluate the correlation between immune cells and DEGs.

### Real-time polymerase chain reaction (PCR)

Reverse transcriptional PCR was performed using the TaKaRa Ex Taq (Takara) and a TaKaRa PCR Thermal Cycler Dice^®^ Touch (Takara), according to the manufacturer's directions. Real-time quantitative PCR was performed using a TAKARA SYBR green Quantitative PCR kit. The relative expression of each gene was determined using the 2-ΔΔCt technique and normalized to the expression of the housekeeping gene glyceraldehyde 3-phosphate dehydrogenase. The gene-specific primers (Huayin, Guangzhou, China) are listed in Table S1.

### Statistical analysis

Statistical analyses were performed using R (version 4.3). Statistical significance was set at p < 0.05 in all tests.

## Results

### Clinical characteristics of the patients

**Fig. 1:**
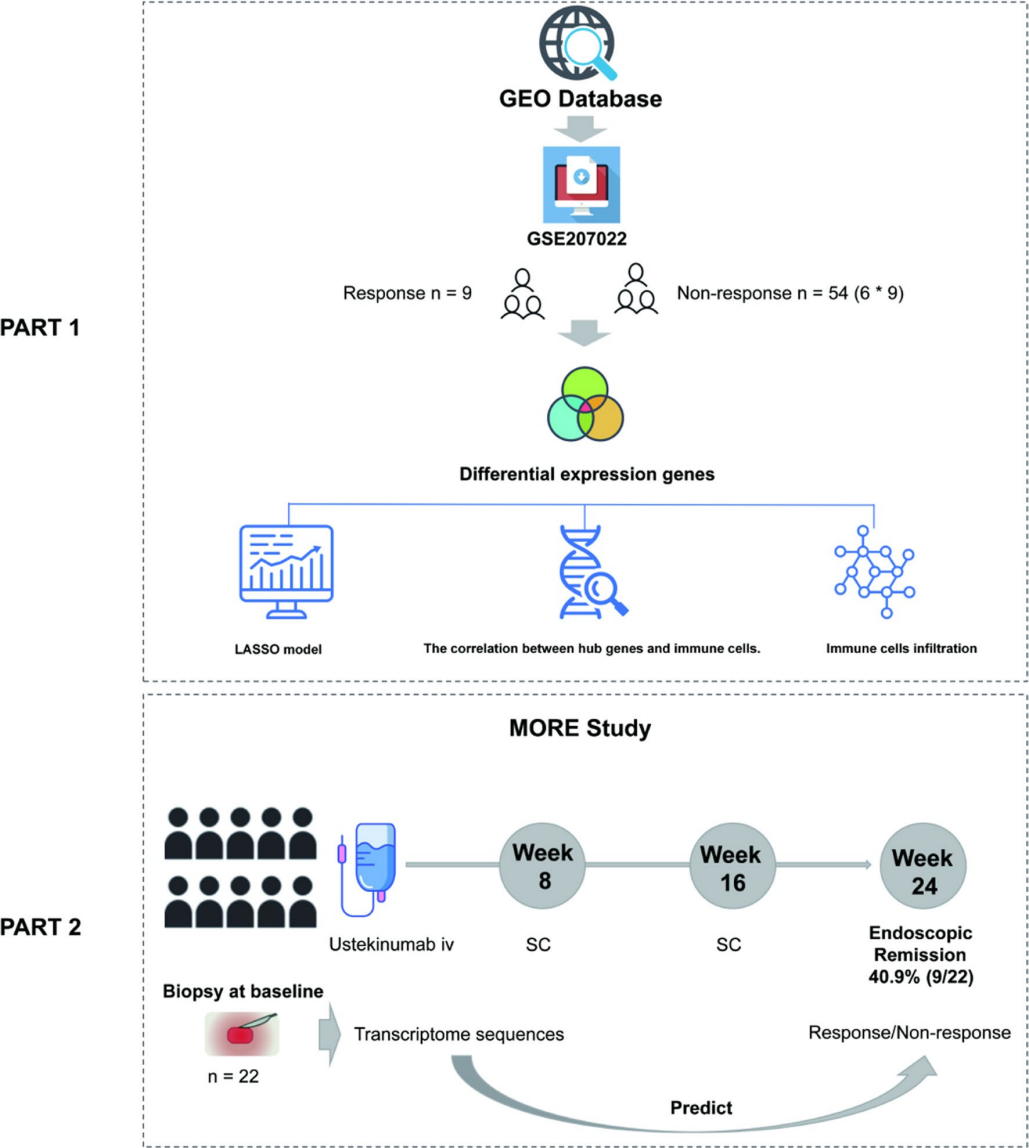
The study workflow. Integrating bioinformatics analysis and machine learning, two gene markers were developed and validated to predict treatment response

The GSE207022 dataset was used as a discovery dataset. Twenty-two patients with active CD were included in the validation cohort. All twenty-two patients underwent colonoscopy at week 24, and 9 (40.9%) achieved endoscopic remission. The clinical characteristics of the patients are shown in Table S2. The baseline demographic characteristics were similar between the response and nonresponse groups. The study flowchart is presented in Fig. 1.

### Gene expression differences in UST response vs non-response patients

**Fig. 2:**
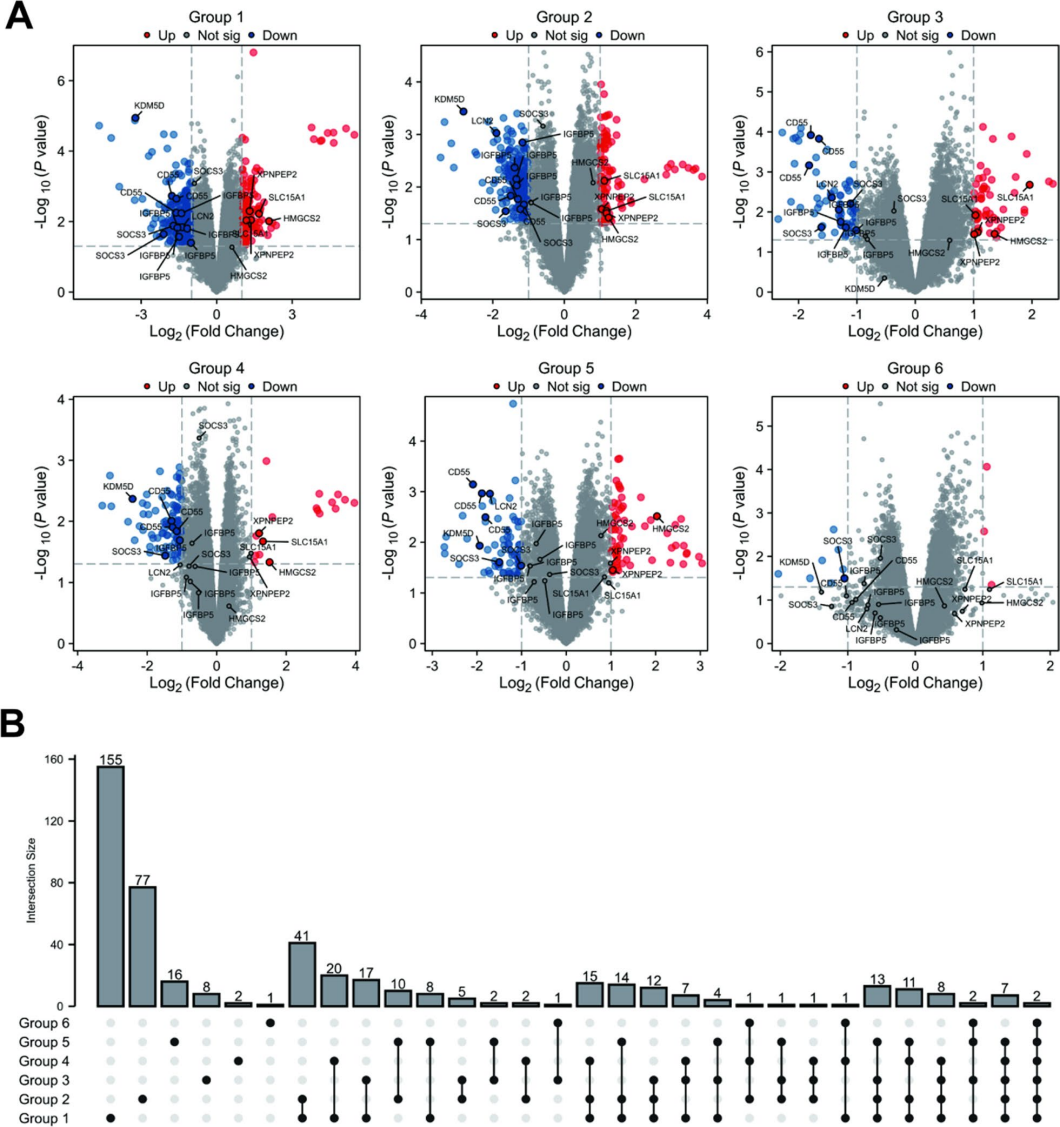
Characteristics of significant differentially expressed genes in ustekinumab response. **A** Volcanic distribution plot of differential genes. Differential genes in response samples comparable to those in nonresponse samples. **B** UpSet plot showing the number of differential genes in each group and those shared by the groups. The number above each column represents the intersection size of differential genes. The connected dots represent the common differential genes across connected cohorts. The response and nonresponse groups included 9 samples

In order to ensure that each group has a similar distribution of samples, the consistency of the within-group samples in non-response arm was analyzed by PCA-Class analysis. The results showed no clear separation between groups (Figure S1). The most significant DEGs were screened out in each group (Fig. 2A). A gene was considered significantly differential expressed when it existed in greater than or equal to 3 groups. Finally, we found 99 genes with significant expression levels between response arm and non-response arm by limma differential analysis (Fig. 2B).

### Functional enrichment of DEGs

**Fig. 3:**
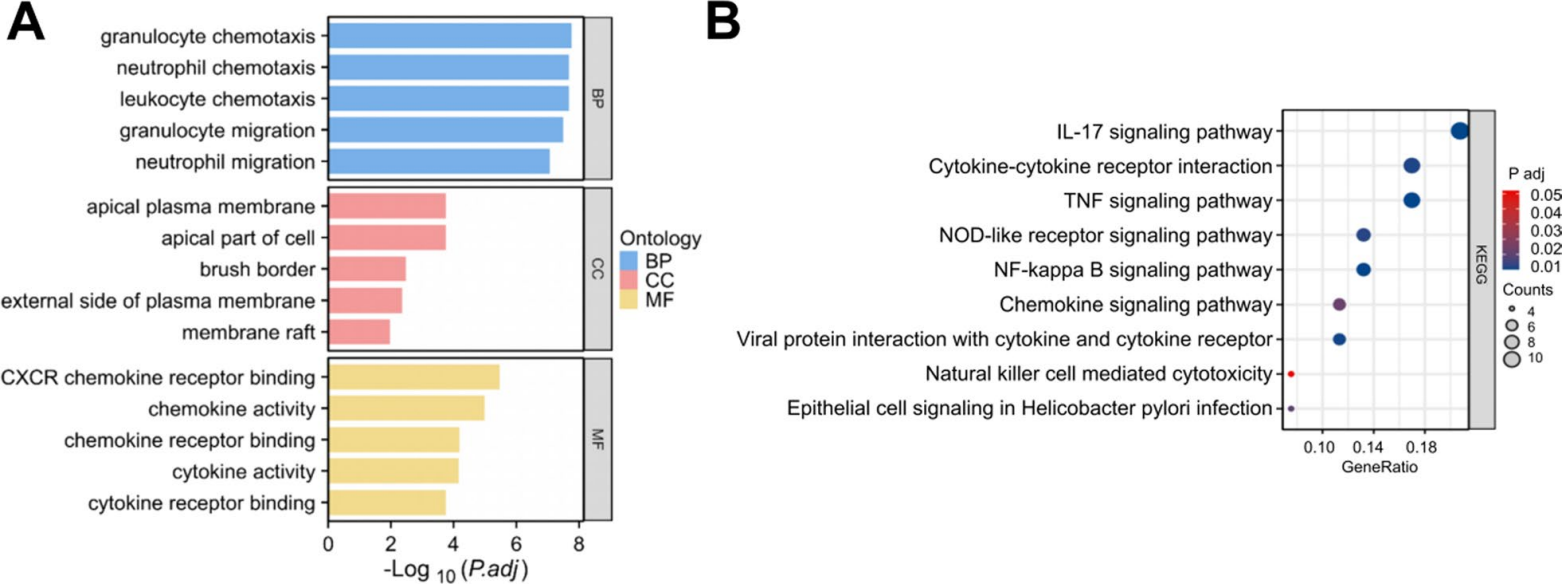
Functional enrichment of differential genes and construction of protein interaction networks. **A** GO analysis results signaling pathways associated with the enriched differential genes. **B** KEGG analysis results of signaling pathways associated with the enriched differential genes

Differential gene-related signaling pathways were explored using GO and KEGG enrichment analyses, which revealed significant enrichment in pathways such as cytokine activity, chemokine receptor binding, and cytokine receptor binding in the GO analysis (Fig. 3A). In addition, KEGG analysis showed significant enrichment in the TNF signaling pathway, NOD-like receptor signaling pathway, and Cytokine-cytokine interaction (Fig. 3B).

### LASSO model to identify potential predictive markers

**Fig. 4:**
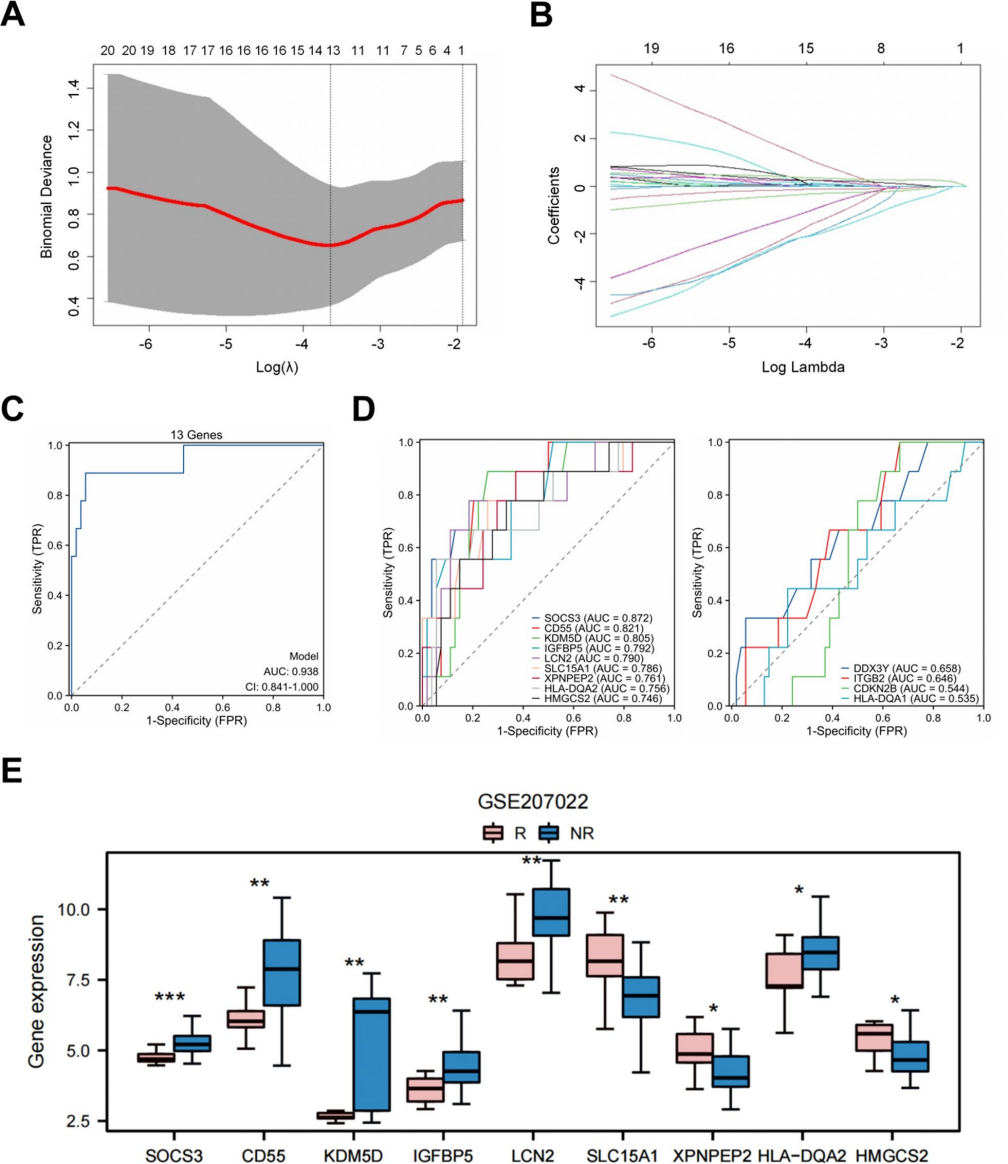
Features selection using the LASSO algorithm and evaluation. **A** Selection of the tuning parameter (Lambda) in the LASSO model using tenfold cross-validation. **B** LASSO coefficient profile of the 10 texture features. A vertical line is drawn at the optimal value selected using the tenfold cross-validation process in (**A**). The 13 features with non-zero coefficients are included to construct the signature. **C** The AUC plot shows the diagnostic efficacy of the LASSO model in predicting response to ustekinumab. *AUC* area under the curve. **D** The AUC plot shows the diagnostic efficacy of each gene in predicting response to ustekinumab.** E** Box plot comparing gene expression between response (n = 9) and non-response (n = 54). *p < 0.05, **p < 0.01, ***p < 0.001

Ninety-nine candidate genes were selected for feature screening using LASSO regression (Fig. 4A, B). We obtained 13 non-zero coefficient characteristics. The results demonstrated strong diagnostic performance of the prediction model based on 13 genes, with an AUC curve of 0.938 (Fig. 4C). Single genes were analyzed for their association with UST response by individually evaluating their ROC characteristics. Figure 4D shows that the genes SOCS3, CD55, KDM5D, LCN2, SLC15A1, XPNPEP2, HLA-DQA2, and HMGCS2 had AUC values > 0.7, while the remaining signature genes had AUC values of 0.5 to 0.7. Nine genes with AUC values > 0.7 were classified as hub genes.

### Univariate logistic regression analysis

We conducted univariate regression analysis on the nine hub genes and visualized the results using a random forest plot. Table 1 shows that SLC15A1 (HR 2.815, p = 0.008), XPNPEP2 (HR 3.455, p = 0.014) and HMGCS2 (HR 3.383, p = 0.027) were better UST response predictors. However, SOCS3 (HR 0.004, p = 0.004), CD55 (HR 0.386, p = 0.010), KDM5D (HR 0.512, p = 0.024), LCN2 (HR 0.375, p = 0.007) and HLA-DQA2 (HR 0.443, p = 0.017) were better predictors of UST nonresponse. Figure 4E shows the expression levels of the nine genes in the response and nonresponse groups.

**Table 1:**
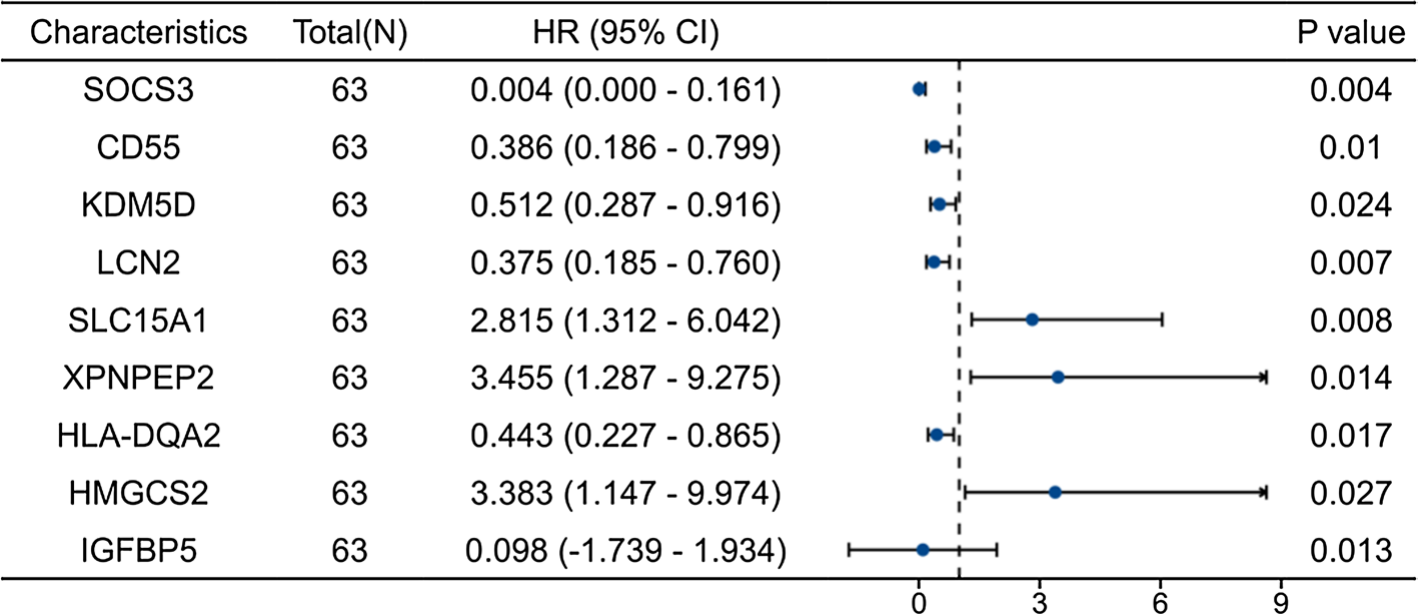
Logistic regression analysis of hub genes in relation to ustekinumab response

### Immune infiltration analysis

**Fig. 5:**
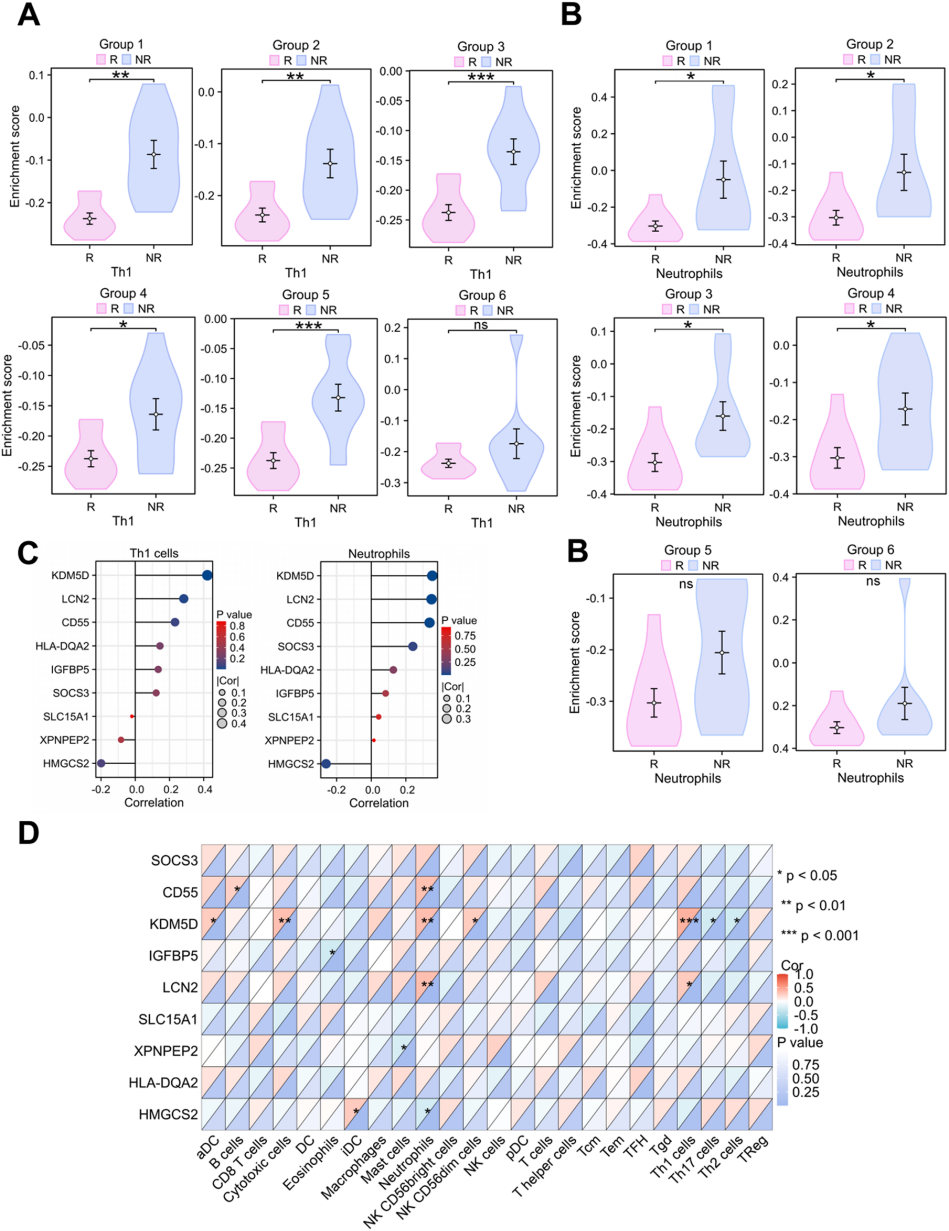
Relationship between key genes of ustekinumab response and immune infltration in Crohn’s disease. **A** Comparison of the Th1 cell fraction between response and nonresponse in six groups. **B** Comparison of neutrophil fractions between response (n = 9) and nonresponse (n = 9) in six groups. **C** Correlation heatmap shows correlation analysis between nine hub genes and 24 immune cell types. **D** Correlation plot shows correlation analysis between nine hub genes and Th1 cells/neutrophils. *p < 0.05, **p < 0.01, ***p < 0.001

The composition of immune cells from intestinal tissues in the response and nonresponse groups was analyzed, and the fractions of 24 immune cells are shown in the boxplot (Figure S2). Group 1 to 5 had significant differences in Th1 cell fractions. More specifically, in nonresponse patients, the proportion of Th1 cells at week 8 was significantly increased compared to that in response patients (Fig. 5A). Futhermore, groups 1–4 had significant differences in the neutrophil and NKCD56dim cells fractions. The neutrophil fraction was relatively high in nonresponse individuals (Fig. 5B). Similarly, group 2 to 5 had significant differences in the T helper cells fractions. Cytotoxic cells, macrophages and Th17 cells were significantly different only in group 1. Intestinal tissue cells did not differ in other immune cell fractions in the response and nonresponse arms. Taken together, Th1 cells may be the main immune cell type that is different between response and nonresponse patients.

We investigated the correlation between immune cells and UST response-related genes. Our results showed that KDM5D was significantly related to the infiltration of various immune cells, including Th1 cells and neutrophils. Notably, LCN2 is also significantly associated with Th1 cells and neutrophils, which are important immune-infiltrating cells associated with UST response (Fig. 5C, D).

### Prospective cohort validation

**Fig. 6:**
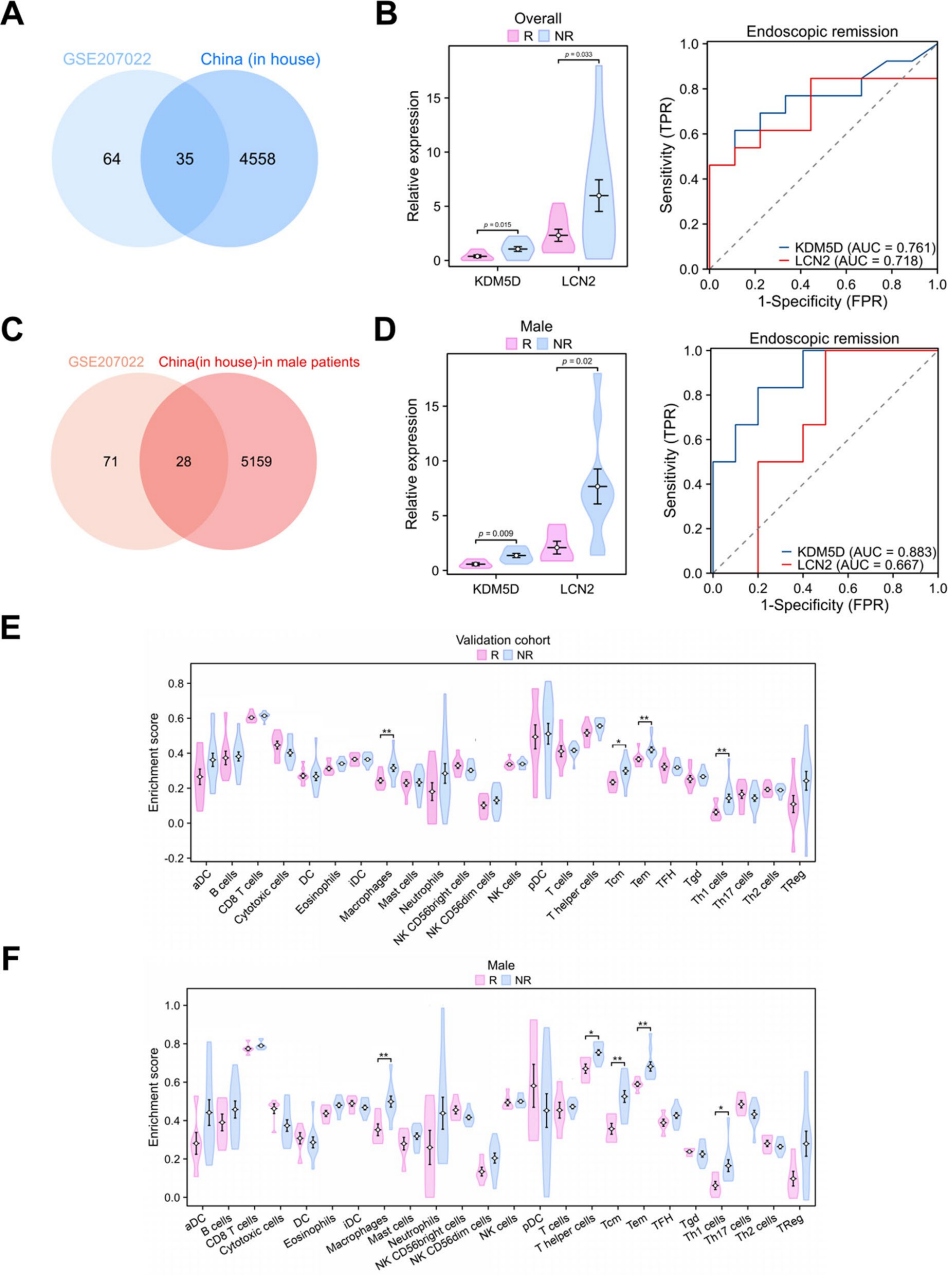
Evaluation of the genes expression and immune infiltration in the dependent cohort. **A** Venn diagrams of the differential genes between the discovery and validation datasets. **B** The relative expression and ROC curves of KDM5D and LCN2 in the validation dataset (R = 9; NR = 13). **C** Venn diagrams of the differential genes between the discovery dataset and male samples (n = 16) of the validation dataset. **D** The relative expression and ROC curves of KDM5D and LCN2 in male samples of the validation dataset (R = 6; NR = 10). **E** Differences in immune cell content between response (n = 9) and nonresponse (n = 13) in the dependent validation cohort. **F** Differences in immune cell content between response (n = 6) and nonresponse (n = 10) in male patients of the dependent validation cohort

Next, we validated our finding in an independent patient cohort. We included an independent dataset of the intestinal tissues of twenty-two patients with CD (male: n = 16; female: n = 6) including 9 response samples and 13 nonresponse samples, as a validation dataset. First, we conducted differential gene expression analysis. Intersectional analysis revealed 35 DEGs (Fig. 6A). As shown in Fig. 6B, the expression levels of both genes in the nonresponse arm were significantly higher than in the response arm. The AUCs for KDM5D and LCN2 were 0.761 and 0.718, respectively.

The KDM5D gene resides on the Y chromosome and is expressed only in males. Therefore, we further analyzed the male samples separately using the same analysis. Intersectional analysis revealed 28 DEGs (Fig. 6C). As shown in Fig. 6D, the expression levels of both genes in the nonresponse arm were significantly higher than in the response arm. The AUCs for KDM5D and LCN2 were 0.883 and 0.667, respectively. We also analyzed female samples separately using the same procedure. Intersection analysis revealed five DEGs: LCN2, PI3, PDZK1IP1, CXCL5 and SIK1 (Figure S3). Thus, these genes may serve as potential biomarkers in female CD patients.

**Fig. 7:**
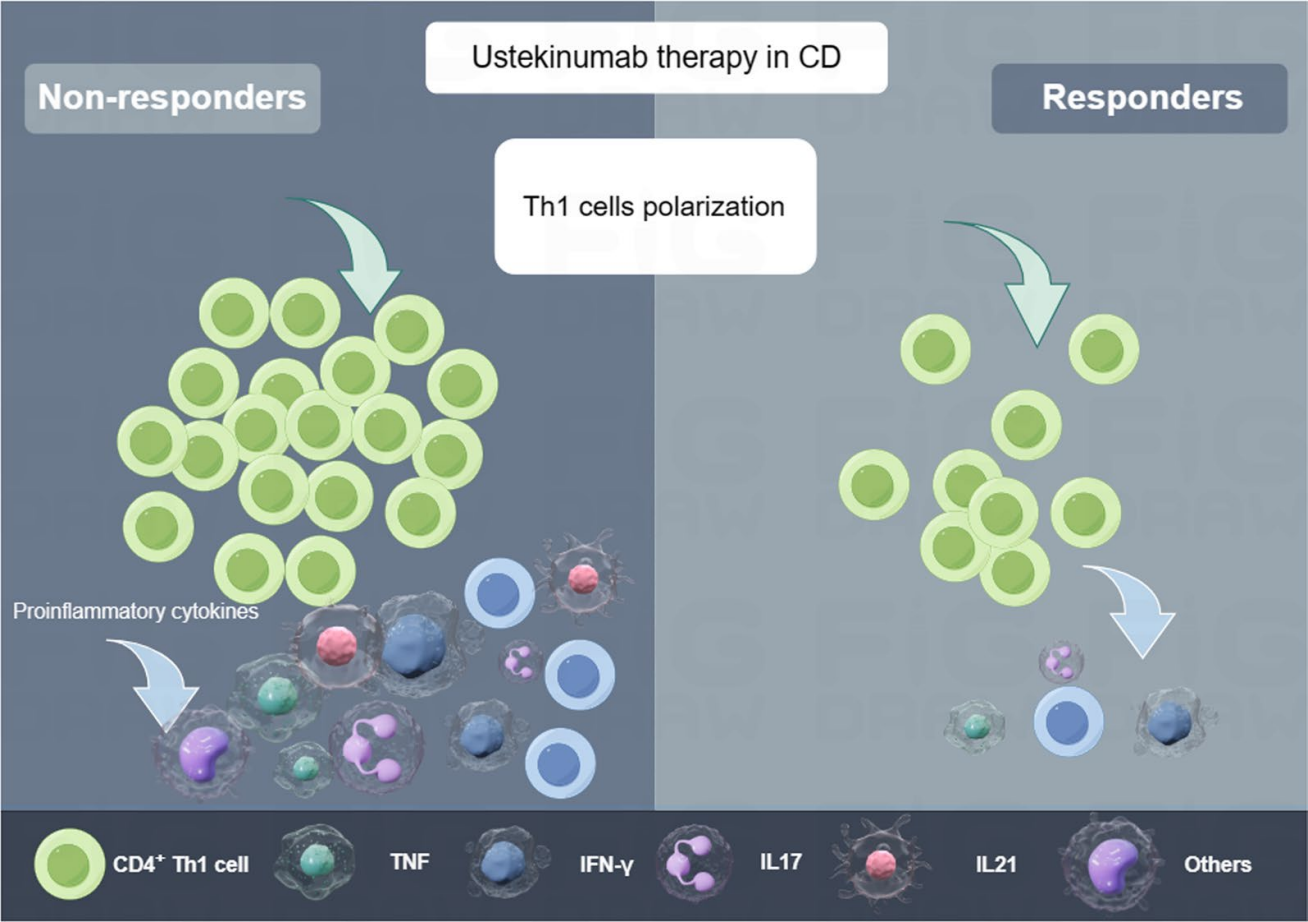
Schematic diagram showing the potential mechanism of Th1 polarization leading to nonresponse to ustekinumab

After performing immune infiltration analysis in the overall cohort and male samples, the results showed that UST response was related to Th1 cells but not neutrophils (Fig. 6E, F), which further confirms the correlation between Th1 polarization and UST nonresponse. Taken together, these data indicate that upregulation of KDM5D and LCN2 could increase infiltration of Th1 cells, which may release inflammatory factors and initiates downstream inflammatory response, leading to a poor initial response. Therefore, we speculate that the upregulation of KDM5D and LCN2 may increase infiltration of Th1 cells and neutrophils, which may release inflammatory factors and initiates downstream inflammatory response (Fig. 7). We also analyzed mRNA expression of KDM5D and LCN2 in intestine. As Figure S4 showed, mRNA expression of KDM5D and LCN2 were significantly higher in the nonresponse arm than in the response arm.

## Discussion

In this study, we found several genes and Th1 polarization were related to response to UST. Among these genes, KDM5D and LCN2 were upregulated in patients with CD who were nonresponsive to UST therapy, which may promote Th1 cell polarization. Additionally, we observed that the effectiveness of UST treatment in male patients was worse than that in female patients (p = 0.038) (Table S3), and the KDM5D, which resides on the Y chromosome, was expressed only in males. Therefore, KDM5D may act as a driver for the significant expansion of Th1 cells to drive gender differences in UST therapy. To our knowledge, it is the first study to predict UST response from the perspective of mucosal mRNA expression.

To date, several studies have explored the relationship between clinical, biological, and pharmacological factors and the initial response to UST. Lower Harvey-Bradshaw Index (HBI) baseline values, female sex and UST trough levels were predictors [29–32]. Dulai et al. [33] created the UST clinical decision support tool (UST-CDST), which was derived from a post hoc analysis of UNITI trials. Using real-world data, the UST-CDST has demonstrated effectiveness in predicting clinical remission and relapse of UST in patients with moderate to severe CD [34]. However, current predictive models rely primarily on general phenotypic, biochemical, and demographic factors, which may lack specificity and reproducibility. Intestinal tissue transcriptomics has shown promise for predicting treatment responses in patients with IBD. In an initial study by Arijs et al. [35], an mRNA microarray was used to identify five highly differentially expressed genes (TNFRSF11B, STC1, PTGS2, IL13RA2, and IL11). These genes were able to accurately predict endoscopic remission in response to infliximab treatment, with a sensitivity of 95% and specificity of 85%. No studies have reported a correlation between intestinal gene expression and endoscopic remission in patients with CD receiving UST therapy. Therefore, in this study, we explored the predictors of nonresponse to UST therapy in patients with CD and externally verified these findings with the goal of improving the cost-effectiveness of medical therapy by avoiding ineffective treatments.

CD is a canonical disease mediated by Th1 cells [36]. Th1 cell differentiation is significantly influenced by IL-12, which further stimulates the production of inflammatory cytokines, including interferon (IFN)-γ, in activated Th1 cells [37, 38]. UST, an anti-IL-12p40 monoclonal antibody proven successful in treating patients with CD, is particularly appealing because it can potentially suppress both Th1 cell types simultaneously [20, 39]. Interestingly, in this study, we observed that nonresponse to UST therapy in patients with CD was associated with a higher Th1 cell fraction. These findings in the discovery and validation cohorts were consistent with Th1 polarization. A prior study found that administering two Fontolizumab doses, an anti-IFN-γ antibody that disrupts Th1 polarization andmacrophage, monocyte, and natural killer cell activation, to patients with active CD led to higher clinical response rates and remission induction compared to placebo [40]. Therefore, excessive Th1 cells activation in some patients with CD may be responsible for nonresponse to UST therapy.

In our study, 13 key genes involved in nonresponse to UST therapy were differentially expressed, including SOCS3, CD55, KDM5D, IGFBP5, LCN2, SLC15A1, XPNPEP2, HLA-DQA2, HMGCS2, DDX3Y, ITGB2, CDKN2B and HLA-DQA1. Futhermore, KDM5D and LCN2, core differential genes, showed a positive correlation between gene expression and the Th1 cell infiltration in nonresponse patients. LCN2, also known as siderocalin or neutrophil gelatinase–associated lipocalin (NGAL), is a powerful bacteriostatic protein packed in neutrophil granules that is discharged at inflammatory sites. LCN2 serves as a dependable indicator of illness severity in ulcerative colitis and CD, differentiating between active disease and remission with heightened sensitivity compared to CRP [41, 42]. Two studies have demonstrated that measureing of serum LCN2 levels is useful to determine an appropriate response to anti-TNF α treatment [43, 44]. La Manna et al. [45] detailed the direct effect of LCN2 on CD4 + T cells. An environment rich in pro-inflammatory cytokines, such as IL-12 and IFN-γ, which are crucial for Th1 differentiation, can be promoted by the role of LCN2 in innate immunity. LCN2 may directly affect T cells by influencing their metabolism and function and promoting a Th1 response [45, 46]. A previous study indicated that LCN2 has the potential to enhance Th1 cell differentiation via the IL-12/STAT4 pathway, either in an autocrine or paracrine manner [46]. This supports our discovery that the LCN2 upregulation leads to Th1 cell differentiation. Since the KDM5D gene is located on the Y chromosome in humans, we wanted to determine whether this gene affects the association between sex and the response of patients with CD to UST. Feagan et al. [18] and Laura et al. [29] reported worse responses to UST in male patients with CD [30]. A clinical study at our center also demonstrated that the UST treatment effectiveness in male patients with active CD who had a disease duration greater than 2 years was worse than that in female patients (*p* = 0.038) [31]. Therefore, KDM5D may drive sex differences in patients with CD receiving UST therapy as a driver of significant Th1 cell expansion.

Sex differences in response to UST therapy for CD are influenced by a complex interplay of genetic immunological factors, as well as hormonal differences [47, 48]. A significant reduction in estrogen receptor β expression has been observed in colonic mucosa from patients with active CD compared with those in remission [48]. KDM5D is encoded on the Y chromosome, which may cause the sex difference in response to UST. However, whether KDM5D has a cross-talk with other factors as hormone remains unclear. This approach underscores the importance of personalized medicine in optimizing treatment strategies for both male and female patients with CD. As mentioned above, a worse response to UST has been reported in male patients with CD than in female patients [29–31]. According to our results, KDM5D (Lysine Demethylase 5D), a member of the JARID1 family of histone demethylases that specifically removes tri- and di-methyl groups from histone H3 lysine 4 (H3K4me3/2), has emerged as the sole Y-chromosome gene with differential expression between response and nonresponse to UST therapy in human males. KDM5D influences Th1 cell polarization by altering the epigenetic landscape [49–51]. Together, these data support the view that KDM5D upregulation drives worse outcomes in males with CD receiving UST therapy. However, further studies are required to assess the contribution of KDM5D to the induction Th1 polarization. This underscores the viability of LCN2 and KDM5D as key targets for nonresponse to UST therapy. Interestingly, we also found that there was sex difference in response to UST, which need to be clarified in the future study. These insights underscore the importance of personalized medicine approaches that consider individual genetic, epigenetic, and gender-specific factors to optimize treatment strategies for patients with CD. Finally, we analyzed the changes in LCN2 and KDM5D mRNA expression by quantitative real-time PCR to prove that they were harmful to patients with CD who respond to UST therapy. We intend to conduct further research to elucidate the underlying molecular mechanism in upcoming studies.

We identified two new key genes related to the response of UST therapy in patients with CD, which may be new biomarkers for nonreponse to UST. The role of LCN2 in IBD has been extensively studied. Its overexpression is directly associated with the induction of Th1 differentiation. Another gene, KDM5D, may drive sex differences in patients with CD receiving UST therapy, as a driver of significant Th1 cell expansion. However, our study had some limitations. Firstly, the sample size extracted from the database was small. Studies with larger sample size is necessary to confirm the valuable initial insights. Secondly, in vitro and in vivo experimental validation for the findings are lacking. Further studies will involve cell function experiments involving overexpression or knockdown of LCN2 and KDM5D, as well as animal experiments using a mouse model of DSS-induced colitis. Despite these limitations, the preliminary findings offer valuable insights.

## Conclusions

In conclusion, bioinformatics analysis and external validation showed that Th1 cells play critical roles in the nonresponse to UST therapy of patients with CD. Moreover, we identified KDM5D and LCN2 as promising candidates for predicting response to UST therapy in patients with CD. Our findings provided evidence that Th1 cells polarization could be a potential predictor for UST nonresponse in patients with CD, which could facilitate the establishment of novel approaches to alleviate the disease burden.

### Supplementary Information

Supplementary material 1.

Supplementary material 2.

Supplementary material 3.

Supplementary material 4.

Supplementary material 5.

## Data Availability

The datasets used and analyzed during the current study are available from the corresponding author on reasonable request.

## Abbreviations

AUC: Area under the curve
CD: Crohn’s disease
CI: Confidence interval
DEGs: Differentially expressed genes
GEO: Gene expression omnibus
GO: Gene ontology
GWAS: Genome-wide association study
HR: Hazard ratio
IBD: Inflammatory bowel disease
IFN: Interferon
IL: Interleukin
KEGG: Kyoto encyclopedia of genes and genomes
LASSO: Least absolute shrinkage and selection operator
OSM: Oncostatin M
OSMR: Oncostatin M receptor
NGAL: Neutrophil gelatinase–associated lipocalin
PCA: Principal component analysis
PCR: Polymerase chain reaction
ROC: Receiver operating characteristic
SES-CD: Simplified endoscopic score of CD
ssGSEA: Single-sample gene set enrichment analysis
Th1: T helper cells type 1
TNF: Tumor necrosis factor
UST: Ustekinumab
UST-CDST: UST Clinical Decision Support Tool
