# Supplementary figures and images for: Intestinal mRNA expression profiles associated with mucosal healing in ustekinumab-treated Crohn's disease patients: bioinformatics analysis and prospective cohort validation
Source: J Transl Med. 2024 Jun 26;22:595. doi: 10.1186/s12967-024-05427-w (PMC11210135; doi:10.1186/s12967-024-05427-w)

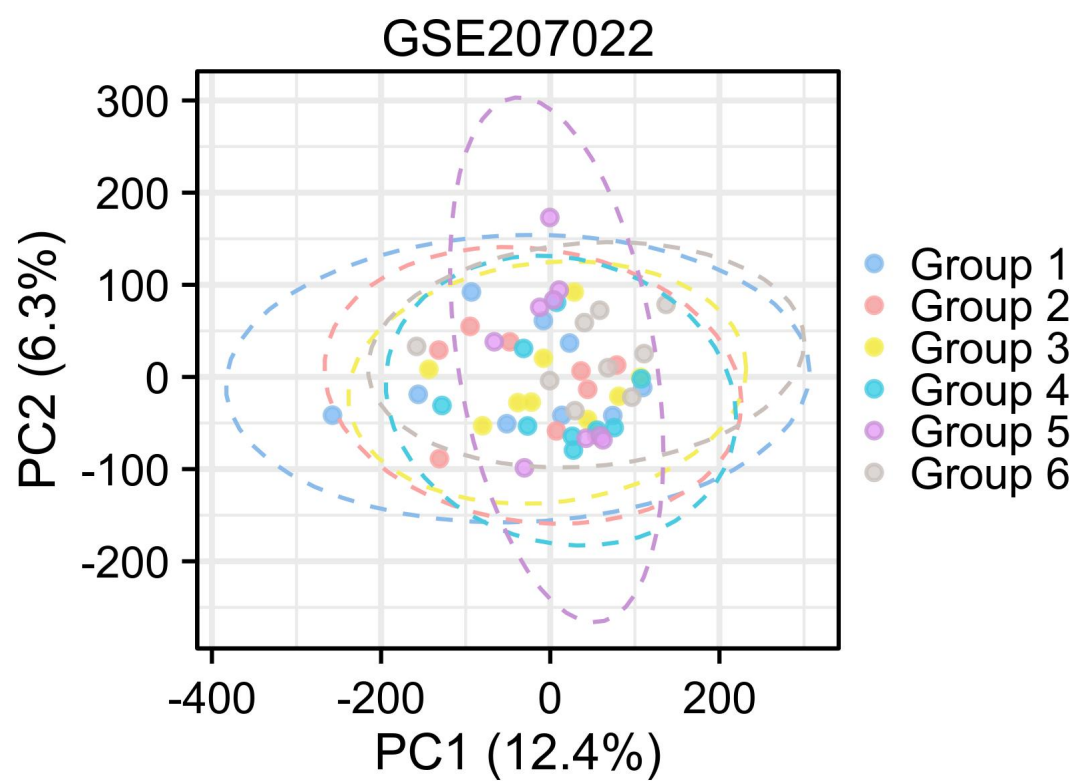

**Figure S1.** Principal component analysis (PCA) plot showing 54 samples in 6 groups.

Supplement: Supplementary file 1 — Supplementary material 1. [file 12967_2024_5427_MOESM1_ESM.pdf]

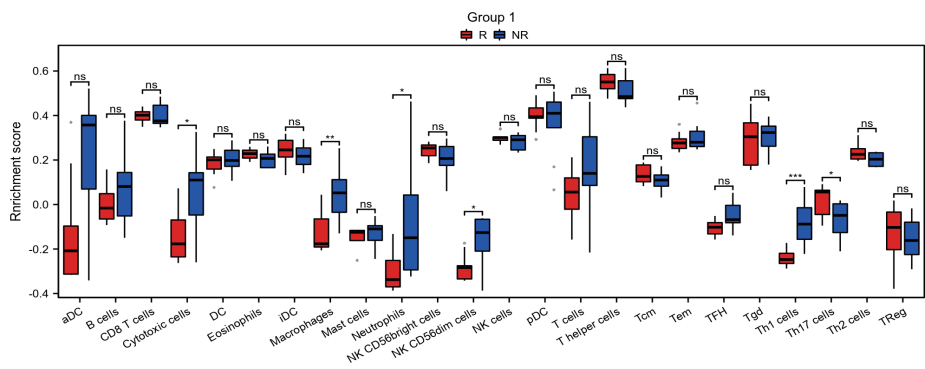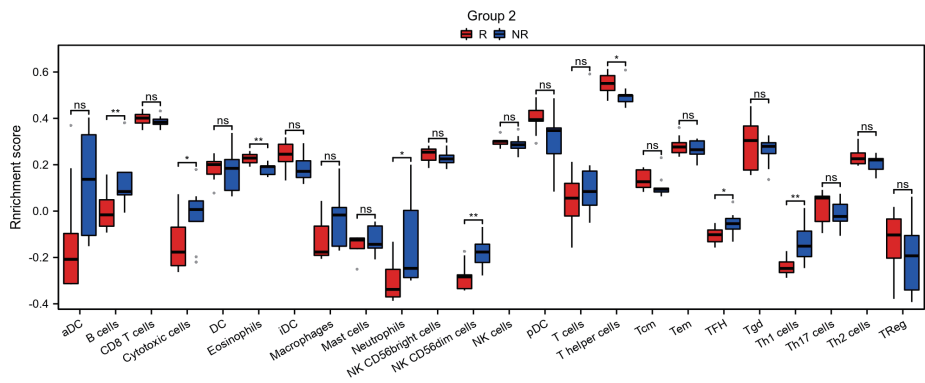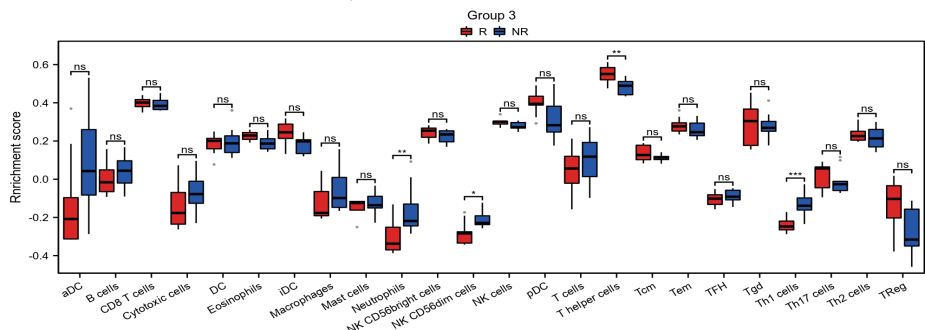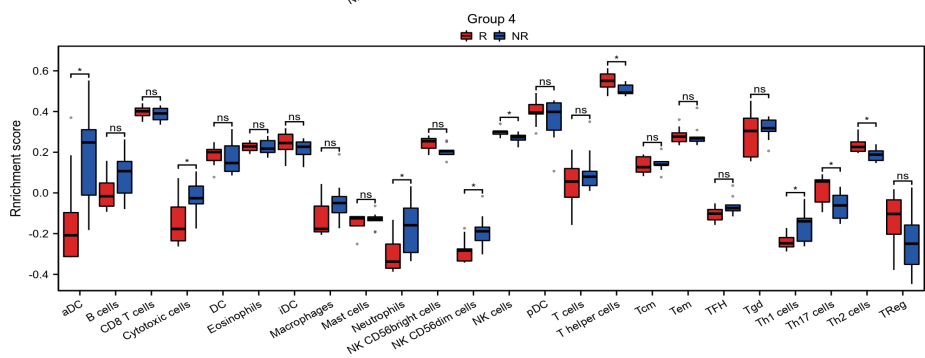

Supplement: Supplementary file 2 — Supplementary material 2. [file 12967_2024_5427_MOESM2_ESM.pdf]
