# Supplementary material for: Intestinal mRNA expression profiles associated with mucosal healing in ustekinumab-treated Crohn's disease patients: bioinformatics analysis and prospective cohort validation
Source: J Transl Med. 2024 Jun 26;22:595. doi: 10.1186/s12967-024-05427-w (PMC11210135; doi:10.1186/s12967-024-05427-w)
Supplement: Supplementary file 3 — Supplementary material 3. [file 12967_2024_5427_MOESM3_ESM.pdf]

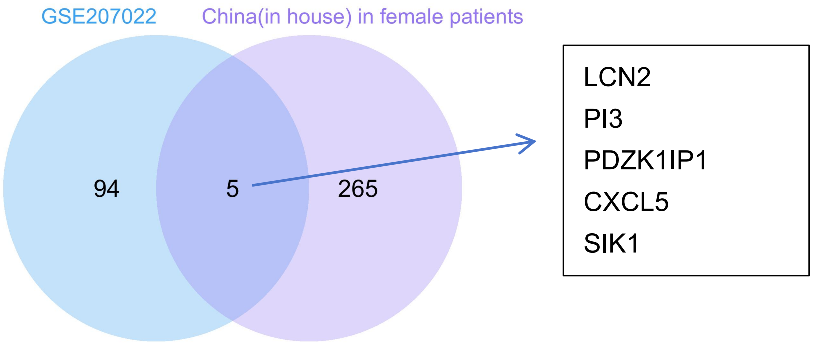

Figure S3. Venn diagrams of the differential genes between the discovery dataset and female samples (n = 6) of the validation dataset.
