# Supplementary material for: Intestinal mRNA expression profiles associated with mucosal healing in ustekinumab-treated Crohn's disease patients: bioinformatics analysis and prospective cohort validation
Source: J Transl Med. 2024 Jun 26;22:595. doi: 10.1186/s12967-024-05427-w (PMC11210135; doi:10.1186/s12967-024-05427-w)
Supplement: Supplementary file 4 — Supplementary material 4. [file 12967_2024_5427_MOESM4_ESM.pdf]

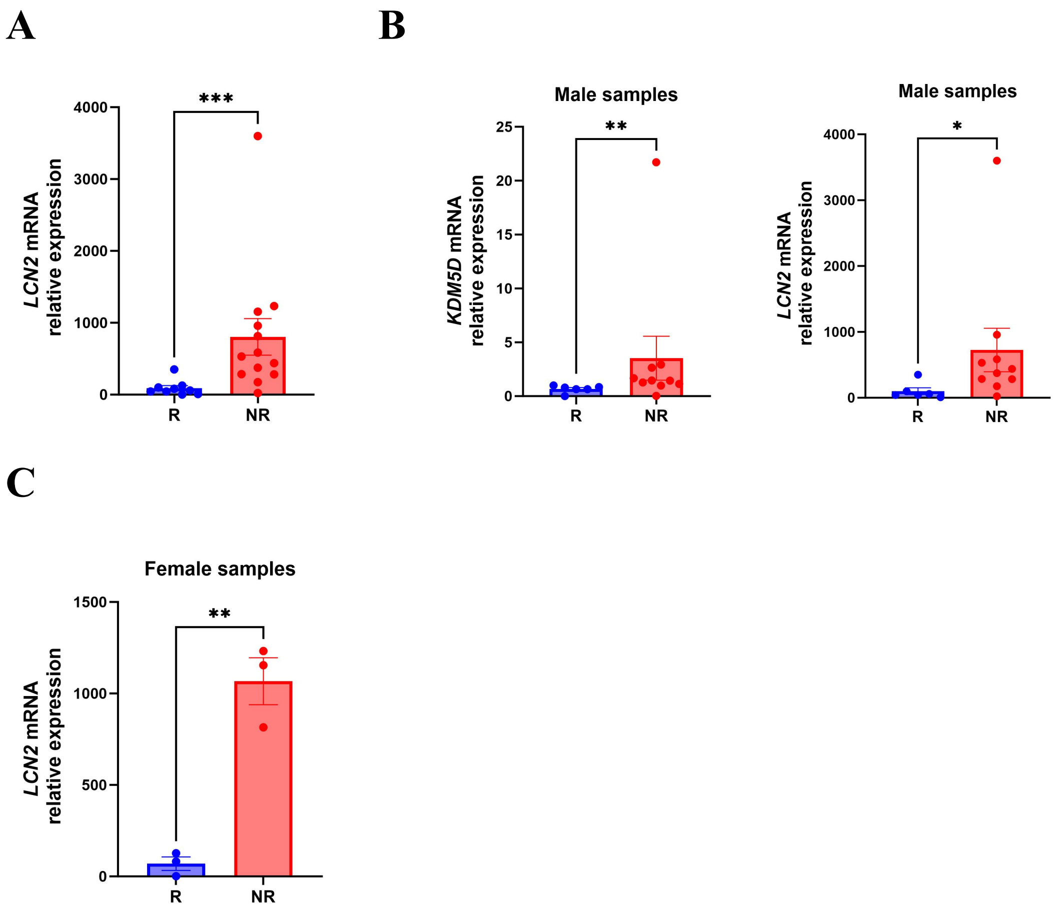

Figure S4. (A) Relative expression of LCN2 mRNA analysed using qRT-PCR in the validation dataset (R = 9; NR = 13). (B) Relative expression of KDM5D and LCN2 mRNA analysed using qRT-PCR in male samples of the validation dataset (R = 6; NR = 10). (C) Relative expression of LCN2 mRNA analyzed using qRT-PCR in female samples of the validation dataset (R = 3; NR = 3). $^*p < 0.05$, $^{**}p < 0.01$, $^{***}p < 0.001$.
