# Supplementary material for: Intestinal mRNA expression profiles associated with mucosal healing in ustekinumab-treated Crohn's disease patients: bioinformatics analysis and prospective cohort validation
Source: J Transl Med. 2024 Jun 26;22:595. doi: 10.1186/s12967-024-05427-w (PMC11210135; doi:10.1186/s12967-024-05427-w)
Supplement: Supplementary file 5 — Supplementary material 5. [file 12967_2024_5427_MOESM5_ESM.docx]

Supplementary data

**Table S1.** The primer sequences [5’to 3’] used in qRT-PCR.

**Table S2**. Clinical characteristics of the independent validation cohort.

**Table S3.** Predictive factors for ustekinumab-induced steroid-free remission at Week 26 in patients who were active Crohn’s disease and had disease duration greater than 2 years.

| **Supplementary Table 1.** The primer sequences [5’to 3’] used in qRT-PCR. | | | |
| --- | --- | --- | --- |
| Gene |  | Forward Primer | Reverse Primer |
| Human | KDM5D | GCGAAAATAAGGCCCATAGCAG | GCCTCCAGTTCATTTAGCCTTTG |
|  | LCN2 | GACAAAGACCCGCAAAAGATGTATG | GCTGGCAACCTGGAACAAAAG |

| **Supplementary Table** **2.** Clinical characteristics of the independent validation cohort. | | | |
| --- | --- | --- | --- |
| Characteristics | R (n=13) | NR (n=9) | *P* value |
| Gender, male, n (%) | 3 (23.1%) | 3 (33.3%) | 0.655 |
| Age, median (IQR) | 24 (22, 35) | 25 (24, 30) | 0.421 |
| Course of disease, month, median (IQR) | 3 (1, 20) | 3 (1, 9) | 0.756 |
| Smoke, n (%) | 0 (0%) | 2 (22.2%) | 0.156 |
| Montreal Classification |  |  |  |
| Location, n (%) |  |  | 0.079 |
| 1 | 3 (23.1%) | 6 (66.7%) |  |
| 2 | 1 (7.7%) | 0 (0%) |  |
| 3 | 9 (69.2%) | 3 (33.3%) |  |
| Behaviour, n (%) |  |  | 1.000 |
| 1 | 7 (53.8%) | 4 (44.4%) |  |
| 2 | 5 (38.5%) | 4 (44.4%) |  |
| 3 | 1 (7.7%) | 1 (11.1%) |  |
| Perianal disease, n (%) | 6 (46.2%) | 5 (55.6%) | 1.000 |
| Extraintestinal manifestation, n (%) | 2 (15.4%) | 1 (11.1%) | 1.000 |
| Anti-TNF α, n (%) | 4 (30.8%) | 2 (22.2%) | 1.000 |
| CRP, median (IQR) | 5.8 (0.9, 13.4) | 32.3 (8.2, 36.6) | 0.051 |
| ESR, median (IQR) | 10 (4, 21) | 16 (10, 23) | 0.524 |
| ALB, mean ± SD | 33.1 ± 3.0 | 35.3 ± 6.8 | 0.392 |
| SES-CD (0w), median (IQR) | 7 (4, 8) | 10 (5, 10) | 0.164 |
| SES-CD (24w), mean ± SD | 2 ± 1.2 | 8.9 ± 3.1 | < 0.001 |

| **Supplementary Table 3.** Predictive factors for ustekinumab-induced steroid-free remission in patients who were active Crohn’s disease and had disease duration greater than 2 years at Week 26. | | | | | | |
| --- | --- | --- | --- | --- | --- | --- |
| Predictors of steroid-free remission at week 26 | Univariate analysis | | | Multivariate analysis | | |
|  | OR | 95%CI | P | OR | 95%CI | P |
| male | 1.854 | 0.991-3.467 | 0.053 | 2.065 | 1.043-4.089 | 0.037* |
| age | 0.689 | 0.971-1.020 | 0.689 |  |  |  |
| smoke | 2.185 | 0.757-6.306 | 0.148 |  |  |  |
| location L2 vs. L1 | 1.067 | 0.334-3.402 | 0.913 |  |  |  |
| location L3 vs. L1 | NA | NA | 1.000 |  |  |  |
| behavior B2 vs. B1 | 0.562 | 0.272-1.158 | 0.118 |  |  |  |
| behavior B3 vs. B1 | 0.708 | 0.309-1.624 | 0.415 |  |  |  |
| active fistula | 2.529 | 1.150-5.560 | 0.021* | 1.802 | 0.773-4.023 | 0.173 |
| perianal disease | 1.161 | 0.638-2.111 | 0.625 |  |  |  |
| bio-naive | 0.702 | 0.355-1.388 | 0.309 |  |  |  |
| previous EEN | 0.945 | 0.520-1.719 | 0.854 |  |  |  |
| previous steroids | 1.386 | 0.750-2.561 | 0.298 |  |  |  |
| previous immunosuppressants | 1.142 | 0.627-2.081 | 0.664 |  |  |  |
| CRP at baseline | 1.019 | 1.005-1.034 | 0.008* | 1.018 | 1.002-1.033 | 0.022* |
| ALB at baseline | 0.961 | 0.904-1.020 | 0.193 |  |  |  |
| CDAI at baseline | 1.008 | 1.002-1.014 | 0.006* | 1.006 | 1.001-1.012 | 0.028* |
| EEN combined at baseline | 2.543 | 1.028-6.291 | 0.043* | 2.538 | 0.955-6.751 | 0.062 |
| Steorids combined at baseline | 1.010 | 0.164-6.198 | 0.992 |  |  |  |
| immunosuppressants at baseline | 1.083 | 0.340-3.453 | 0.893 |  |  |  |
